# Novel method for action potential measurements from intact cardiac monolayers with multiwell microelectrode array technology

**DOI:** 10.1038/s41598-019-48174-5

**Published:** 2019-08-15

**Authors:** Heather B. Hayes, Anthony M. Nicolini, Colin A. Arrowood, Stacie A. Chvatal, David W. Wolfson, Hee Cheol Cho, Denise D. Sullivan, Jérome Chal, Bernard Fermini, Mike Clements, James D. Ross, Daniel C. Millard

**Affiliations:** 1 Axion Biosystems, Inc, Atlanta, GA USA; 2 Emory University, Atlanta, GA USA; 3 Coyne Scientific, Atlanta, GA USA

**Keywords:** Electrophysiology, Cardiovascular biology

## Abstract

The cardiac action potential (AP) is vital for understanding healthy and diseased cardiac biology and drug safety testing. However, techniques for high throughput cardiac AP measurements have been limited. Here, we introduce a novel technique for reliably increasing the coupling of cardiomyocyte syncytium to planar multiwell microelectrode arrays, resulting in a stable, label-free local extracellular action potential (LEAP). We characterized the reliability and stability of LEAP, its relationship to the field potential, and its efficacy for quantifying AP morphology of human induced pluripotent stem cell derived and primary rodent cardiomyocytes. Rise time, action potential duration, beat period, and triangulation were used to quantify compound responses and AP morphology changes induced by genetic modification. LEAP is the first high throughput, non-invasive, label-free, stable method to capture AP morphology from an intact cardiomyocyte syncytium. LEAP can accelerate our understanding of stem cell models, while improving the automation and accuracy of drug testing.

## Introduction

Electrical activation of cardiomyocytes initiates and controls downstream contraction of the human heart. As such, accurate model systems of functional cardiac electrophysiology serve an important role in identifying mechanisms and potential therapies for cardiac dysfunction. Primary cardiomyocytes harvested from animal models have been, and continue to be, a useful model of cardiomyocyte electrophysiology^1,2^, while the advent of human induced pluripotent stem cell-derived cardiomyocytes (hiPSC-CMs) has facilitated the recapitulation of healthy and diseased human biology *in vitro*^3–5^. Although techniques for measurement of cardiac electrophysiology depend on the model system, direct measurement of the cardiomyocyte action potential (AP) with manual patch clamp has been the gold standard for reduced, cell-based preparations.

A key advantage of cell-based assays is the possibility for increased assay throughput due to the simplicity and low-cost of such preparations. The manual patch clamp assay, however, requires significant technical expertise and is limited to a single cell measurement at a time, thus restricting its throughput. Numerous techniques have been introduced in an attempt to produce high quality measurements of the cardiac AP from cell-based preparations at increased assay throughput. Instrumentation for high throughput automated patch clamp (APC) enables ion channel measurements from overexpression systems^6^, but has proven inefficient at capturing AP measurements from cardiomyocytes due to insufficient sealing resistance^7,8^. By comparison, various optical methods produce quality AP measurements^9–12^, but require addition of potentially toxic dyes^13,14^ or genetic editing^15^ and are typically restricted to short measurements of a single sample at a time.

As such, the standard for high throughput *in vitro* multiwell measurements of cardiac electrophysiology has been multiwell microelectrode array (MEA) technology^4,16–19^. Such devices measure the extracellular field potential (FP) from cardiomyocytes adhered to the MEA substrate^20^. The FP derives from the underlying cardiac AP^21,22^, but more closely resembles the clinical electrocardiogram waveform. Cardiomyocyte MEA assays provide a label-free measurement of the cardiac FP from up to 96-wells simultaneously, facilitating their use in drug screening assays^16–19^ or for monitoring long term cardiomyocyte maturation. The difference in shape between the FP and AP, however, can limit or obscure direct comparison to gold standard manual patch clamp recordings, which has motivated the active development of techniques for AP recordings using MEA technology.

Advanced approaches for measuring AP signals with MEA technology have relied on opening pores in the cell membrane or changing the shape of the microelectrode to increase the coupling coefficient, defined as the ratio between the voltage signals acquired on the MEA device and the transmembrane voltage change produced by the adherent cells^23^. Classical methods of whole-cell patch clamp or perforated patch clamp open pores in the cell membrane, thus significantly reducing the junctional membrane resistance and achieving high coupling coefficients^23^. For MEA devices, electroporation, in which delivering strong electrical currents opens holes in the cell membrane, has recently been used to transiently increase the coupling coefficient and acquire AP signals^24–27^. However, the coupling coefficient rapidly decays as the holes reseal, complicating the execution and limiting the adoption of electroporation for multiwell AP measurements. By comparison, the coupling coefficient may also be increased by enhancing the seal resistance of the adherent cells to the electrode^23^. A number of recent devices have utilized 3-D electrodes to acquire AP signals over longer timescales^28,29^, but no commercial implementations exist on a multiwell scale. Outside of these approaches, published observations indicate that AP measurements may be made from cardiomyocytes attached to planar MEAs that spontaneously and sporadically exhibit high coupling coefficients^22^, presumably due to strong attachment of the cell monolayer to the electrode producing a high sealing resistance. These observations are infrequent, though, preventing the development of a practical and reliable AP assay.

Here, we report a novel technique for reliably increasing the coupling coefficient of adherent cardiomyocyte monolayers on commercially-available multiwell MEA plates, thereby enabling multiwell AP assays. The technique, termed the local extracellular action potential (LEAP) assay, applies electrical signals to the planar microelectrodes to induce the same strong cell coupling occasionally observed spontaneously, resulting in AP signals with high signal-to-noise ratio that are stable over longer assay timescales, label-free, and non-destructive. LEAP was rigorously tested with four hiPSC-derived cardiomyocytes and primary rat cardiomyocytes under various conditions including pacing, compound application, genetic modification, and varying AP morphologies. We demonstrate that LEAP is distinct from traditional electroporation techniques, does not affect the underlying electrophysiology, and agrees with simultaneous measurements of the cardiac FP in the absence and presence of drugs known to affect cardiac electrophysiology. Finally, we describe the advantages of the LEAP assay over existing multiwell FP assays for various applications and clarify boundaries of the assay.

## Results

### LEAP induction transforms a field potential signal to an action potential signal on planar microelectrodes

**Figure 1:**
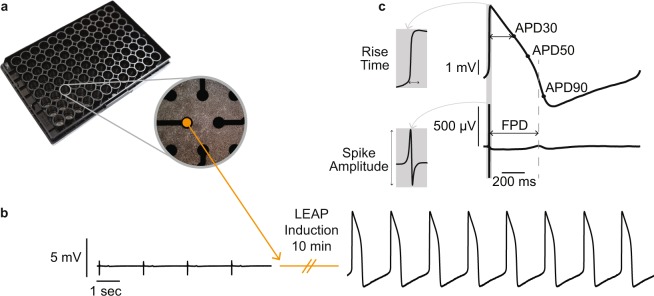
LEAP induction transforms field potentials to action potentials. (**a**) CytoView MEA 96-well plate with inset showing Bright Field 4x Magnification image of iCell CM^2^ cultured in a single well atop eight planar microelectrodes. (**b)** Example signal transformation on a single planar electrode from before and after LEAP induction. (**c)** LEAP and FP waveforms (averaged across 5 beats) recorded from neighboring electrodes in the same well. Markers for depolarization (LEAP: rise time; FP: AMP) and repolarization (LEAP: APD at various stages of repolarization; FP: FPD) are indicated. Gray dashed line highlights that FPD occurs between APD50 and APD90.

When cultured *in vitro*, cardiomyocytes form a spontaneously beating syncytium and produce a cardiac FP signal that may be detected by microelectrodes embedded in the culture plate substrate. An example of a commercially-available transparent multiwell MEA plate seeded with cardiomyocytes is shown in Fig. 1a. This report describes a novel induction process that increases the coupling of the cardiomyocytes to the planar microelectrodes such that the voltage signal detected on the microelectrodes changes from a FP to an AP signal morphology. An example of the voltage signals recorded before and after the induction process is presented in Fig. 1b. Following the induction process, the signal, termed the local extracellular action potential (LEAP), has substantially increased in amplitude and changed in shape to resemble the cardiac AP. The FP and LEAP signals in Fig. 1c were recorded simultaneously from separate electrodes in the same well. The LEAP induction process may operate independently on each electrode (see Experimental Procedures for additional details), thus allowing a direct translation between FP and AP electrophysiology endpoints from the same syncytium.

LEAP signals have been observed spontaneously in previous reports. Here we report at least one spontaneous LEAP signal in ~3.0% of wells (13 out of 432). Spontaneous LEAP signals and induced LEAP signals are functionally equivalent, with each characterized by a large increase in signal strength, a change from a FP to AP signal morphology, and a change in cell-electrode attachment. Supplemental Video S1 illustrates the cell contraction pattern and electrode voltage during a spontaneous LEAP, whereas Supplemental Video S2 presents the cell contraction and electrode voltage before and after LEAP induction. For the remainder of the manuscript, LEAP signals will refer to those generated by the LEAP induction process, as the prevalence of spontaneous LEAP signals is too low and sporadic to warrant assay development.

**Figure 2:**
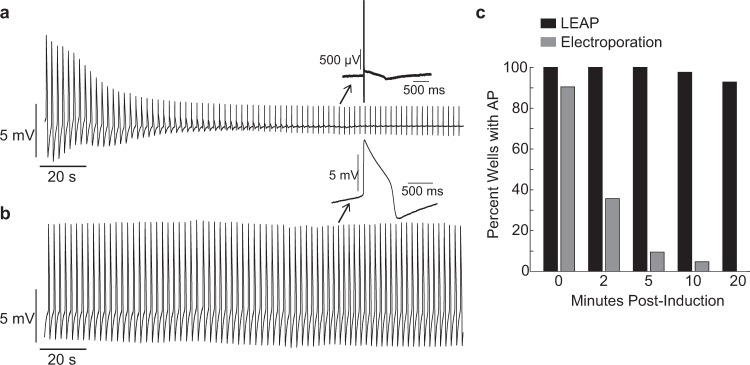
LEAP is stable over long time courses unlike electroporation. Electroporation was applied across 42 wells on a Classic MEA 48-well plate cultured with iCell CM^2^. Following electroporation decay, LEAP was applied to the same wells. (**a)** Representative electroporation signal showing rapid decay back to FP. Inset shows FP shape at two minutes post-induction. (**b)** Representative LEAP signal with stable amplitude. Inset shows stable LEAP shape persists at two minutes post-induction. (**c)** Percentage of wells with a stable AP signal at 0, 2, 5, 10, and 20 minutes post-induction. LEAP persisted for more than 20 minutes, whereas electroporation rapidly decayed.

We compared the stability of LEAP signals to AP signals recorded following electroporation. Electroporation produced AP signals by transiently opening pores in the cell membrane in response to high intensity, short duration electrical stimuli (see Methods). However, the amplitude of the AP signals decayed rapidly over time (see Fig. 2a for example trace), presumably as the membrane pores closed. By comparison, the LEAP signals were stable over physiologically and pharmacologically relevant time scales, as illustrated by the example in Fig. 2b. To quantify signal stability, electroporation was applied to iCell CM2 on all electrodes across 42 wells and the percentage of wells with persistent AP signals was measured at 2, 5, 10, and 20 minutes. In agreement with previous reports^25,26,28^, AP signal amplitude decayed within 2 minutes post-electroporation, and all AP signals had reverted to FPs after 10 minutes. Subsequently, LEAP induction was applied to all electrodes on the same 42 wells. In contrast to electroporation, LEAP signals persisted much longer, with over 90% of wells retaining high amplitude AP signals beyond 20 minutes post-induction (Fig. 2c). Importantly, the value of this technology for screening applications is dependent on reliable measurements from each well on a multi-well plate. For this reason, the experimental protocol (see Methods) for all dosing experiments was designed around the 10–20 minutes of stability in the AP signal afforded by LEAP induction.

**Figure 3:**
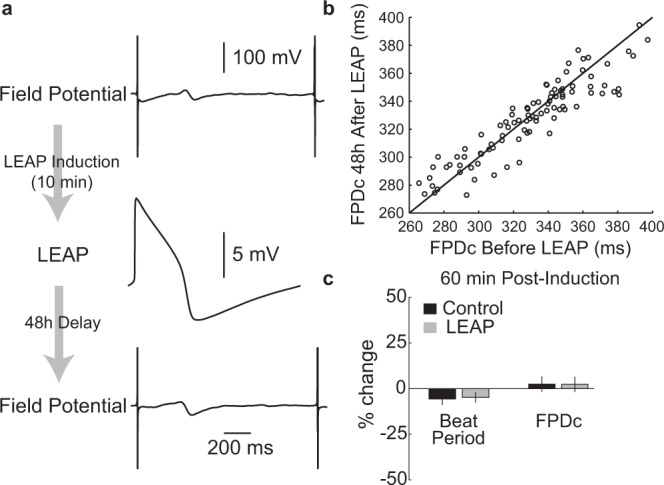
LEAP does not disrupt cardiomyocyte function or behavior. (**a**) FPs were recorded before and 48 hours after LEAP induction on all electrodes of a Classic MEA 96-well plate with iCell CM^2^. The FP was not affected by LEAP induction. (**b)** FPDc before and after LEAP were tightly correlated (R^2^ = 0.84, p < 0.001). Each dot represents the FPDc before and 48 hours after LEAP induction for a well, with the unity line in black for comparison. (**c)** Here, LEAP induction was applied to half of the electrodes in half of the wells on a Classic MEA 96-well plate with iCell CM^2^. FPs were recorded before and 60 minutes after LEAP induction. Bar plots represent the percent change (mean ± standard deviation across wells) in BP and FPDc, measured from the FP signal, from before to 60 minutes post-LEAP induction. Changes in BP and FPD did not differ between control (no LEAP) and LEAP wells (Mann Whitney U-Test, BP p = 0.097, FPDc p = 0.77).

In addition, LEAP induction does not alter or disrupt cell health. FP signals were recorded before LEAP induction across 48 wells plated with iCell CM2. After induction, LEAP signals eventually reverted to FPs, and FPs were recorded 48 hours later. FP shape was unchanged on the same electrode despite successful LEAP induction (Fig. 3a). FPDc before and 48 hours later were tightly correlated across all 48 wells (R2 = 0.84, p < 0.001, Fig. 3b). Further, to show that LEAP is a local effect and does not disrupt neighboring cell behavior, LEAP was induced on half of the electrodes (4) in half of the wells (48) of a 96 well plate. FPs were then recorded from the remaining electrodes in each well before and after LEAP induction. The change in FPD (Mann Whitney U Test, p = 0.77), and BP (p = 0.097) was not different between the LEAP and control wells immediately following LEAP or 1 hour later (Fig. 3c).

### Simultaneous LEAP and FP measurements establish translation between FP and AP signals

**Figure 4:**
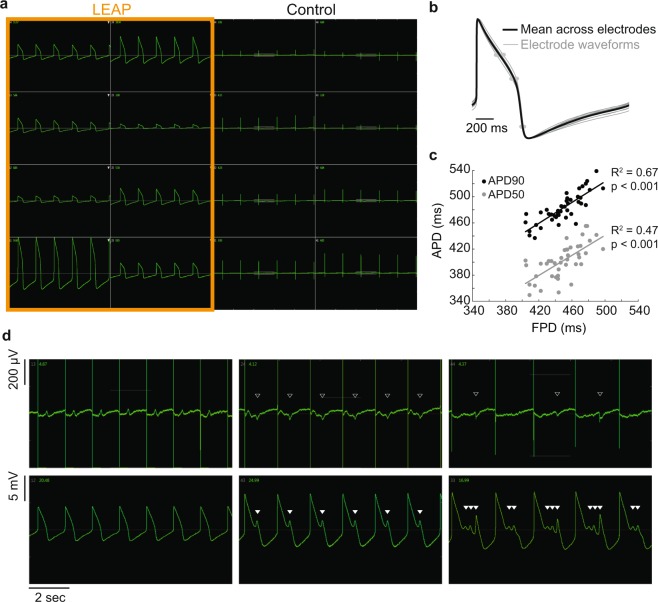
Simultaneous LEAP and FP measurements establish translation between FP and AP signals. LEAP was induced on half of the microelectrodes in each well of a Classic MEA 48-well plate of iCell CM^2^. (**a)** Representative well showing LEAP waveforms from eight electrodes (left) and FPs from eight electrodes (right). (**b)** Despite differences in signal amplitude, LEAP shapes were consistent across electrodes in a well. LEAP waveforms (averaged across 5 beats) from each of eight electrodes are shown in gray with the mean across electrodes overlaid in black. APD30, 50, and 90 are marked with gray dots on each electrode trace. (**c)** APD90 and 50 were correlated with FPD across all wells. Each dot represents the FPD and APD90 (black) or APD50 (gray) from a single well (n = 48 wells). A best fit linear regression is plotted for APD90 (black) and APD50 (gray) as a function of FPD (t-test for slope vs zero, p < 0.001). (**d)** Example FP and LEAP signals from the same well when dosed with DMSO (left) or E-4031 (middle, right). With vehicle control, depolarization and repolarization aligned between the FP and LEAP. With hERG block, both the FP and LEAP were prolonged and EADs developed. EADs were automatically detected on the LEAP signals (filled white triangles) and visually identified on the FP (open white triangles) for direct comparison of features.

LEAP and FP measurements collected simultaneously were compared to define the accuracy of LEAP signals relative to the established technique of multiwell FP measurements. LEAP induction was performed on half of the electrodes per well in a 48-well plate seeded with iCell CM^2^, with an example well presented in Fig. 4a. The amplitude of LEAP signals following induction was typically variable, representing different degrees of cell-electrode coupling, but the AP shape was consistent across electrodes. Figure 4b depicts the individual amplitude normalized LEAP signals (gray, n = 8 electrodes), along with the mean across electrodes (black), from the same electrodes presented in Fig. 4a. The individual LEAP signals were in strong agreement, particularly at APD90, despite sampling from different local populations of cells within the syncytium.

We quantified the automated detection of APD50 and APD90 from the LEAP signals and compared against the semi-automated FPD detection from the control electrodes in each well (Fig. 4c). APD was correlated with FPD across 48 wells (R^2^ = 0.67 for APD90 vs. FPD, p < 0.001; R^2^ = 0.47 for APD50 vs. FPD, p < 0.001), with FPD consistently occurring between APD50 and APD90.

Simultaneous LEAP and FP measurements also directly establish translation of “EAD-like” features in the FP signal with rigorously defined and automatically detected EAD features in the LEAP signal. Figure 4d presents simultaneously acquired FP (top) and LEAP (bottom) signals for control (left, 0.1% DMSO), prolonged (middle, 10 nM E-4031), and severely prolonged (right, 100 nM E-4031) conditions. The EAD features (white triangles) were identified manually in the FP signal and using an automated algorithm for the LEAP signals (see Methods). The EADs in the LEAP signals confirm that the small, negative deflections in the FP signal, often used to mark arrhythmic activity in previous studies^30^, do indeed indicate EAD events. Importantly, the events can be difficult to detect on the FP even by a trained user, as seen on the right. The high signal-to-noise ratio and large amplitude afforded by LEAP induction allow for automated detection of EADs and quantification of associated metrics, such as percentage of beats with EADs.

### LEAP signals reproduce expected responses to ion channel blockers

**Figure 5:**
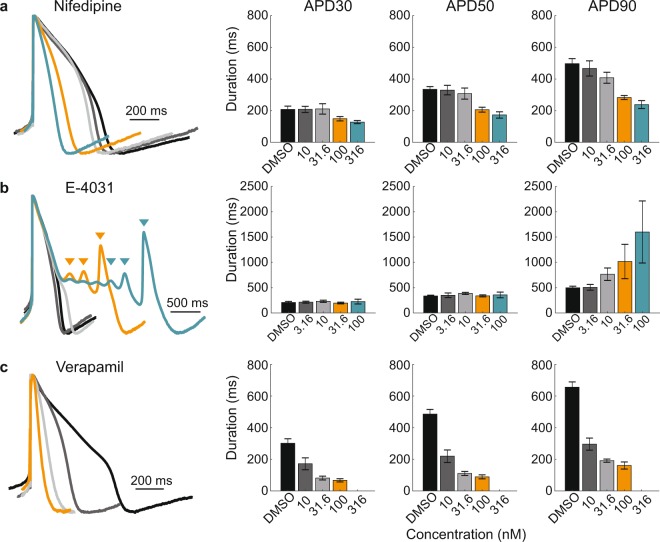
LEAP signals reproduce expected responses to ion channel blockers. Cardiomyocytes (iCell CM^2^) plated on Classic MEA 48-well plates were dosed with vehicle control or one of four doses of Nifedipine (**a**), E-4031 (**b**), and Verapamil (**c**). For each compound, amplitude normalized LEAP waveforms (averaged across 5 beats) from representative wells are overlaid for DMSO (black) and the four increasing concentrations of each compound (dark gray, light gray, orange, and teal). Triangles indicate automatically detected EADs. Bar plots represent the mean ± standard deviation of APD30, 50, and 90 across replicate wells (n = 8 for DMSO, n = 5 for each concentration of Nifedipine and E-4031, n = 3 for each concentration of Verapamil). The highest dose of Verapamil stopped cardiomyocyte beating.

LEAP accurately captures the electrophysiological effects of single and multi-ion channel blockers, identifying drug-induced arrhythmogenic responses as well as more subtle changes in AP shape. To measure drug-induced responses, iCell CM^2^ were dosed with either vehicle control (DMSO, n = 8 wells) or one of four doses of Nifedipine, E-4031, or Verapamil (n = 3–6 wells each) across two Classic MEA 48-well plates. Nifedipine, a calcium channel blocker, induced a dose-dependent shortening of APD (Fig. 5a). In contrast, E-4031, a hERG (Human Ether-a-go-go-related Gene) channel blocker, caused prolongation with increasing doses and induced EADs at the two highest doses (Fig. 5b). Finally, multi-channel effects were also detected. Verapamil blocks hERG potassium channels, but this effect is mitigated by L-type calcium channel block, as reported previously *in vitro*^16^. Thus, Verapamil led to a dose-dependent shortening of APD (Fig. 5c), making verapamil a low arrhythmogenic risk compound^30,31^. At the highest dose (316 nM), Verapamil caused the cells to stop beating.

**Figure 6:**
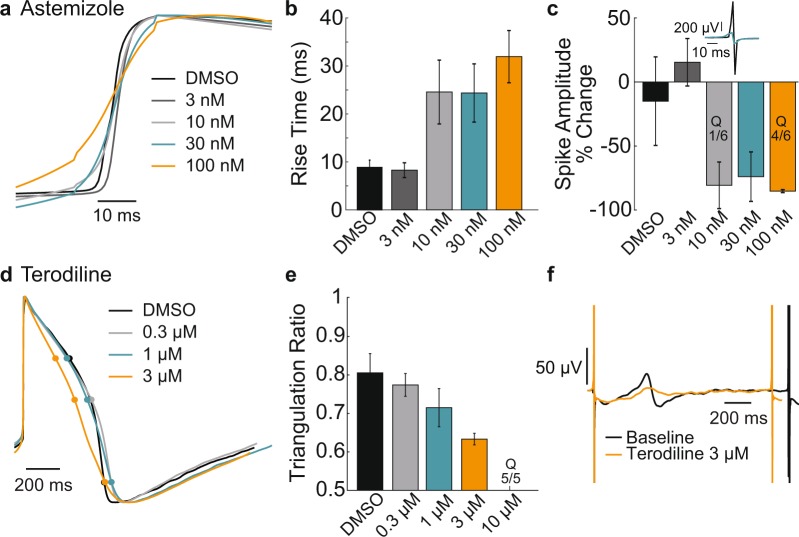
LEAP quantifies rise time and triangulation to reveal subtle changes in action potential morphology. (**a**) Coyne hiPSC-derived cardiomyocytes were plated on a Classic MEA 96-well plate and dosed with vehicle control or one of four doses of Astemizole (**a**,**b**,**c**). The rising phase of LEAP waveforms (averaged across 5 beats) from representative wells are overlaid for DMSO (black) and the four increasing concentrations of Astemizole (dark gray, light gray, teal, orange). Astemizole prolonged the rise time of the AP. (**b)** Bar plots represent the mean ± standard deviation of rise time across replicate wells (n = 13 for DMSO, n = 6 for each concentration of Astemizole). One of six wells became quiescent (Q) in response 100 nM of Astemizole. (**c)** Prolonged rise time corresponded to a reduction in FP AMP, with several wells becoming quiescent (Q) particularly at higher doses. (**d)** iCell CM^2^cardiomyocytes were dosed with vehicle control or one of four doses of Terodiline (**d**,**e**,**f**). LEAP waveforms (averaged across 5 beats) from representative wells are overlaid for DMSO (black) and three increasing concentrations of Terodiline (light gray, teal, orange). Terodiline shutdown beating in all replicates at the fourth and highest dose (Q, 10 µM). (**e)** Bar plots represent the mean ± standard deviation of the triangulation ratio across replicate wells (n = 10 for DMSO, n = 5 for each concentration of Terodiline). (**f)** AP triangulation corresponded to broadening and shrinking of the T-wave of the corresponding FP. FPs (averaged over 5 beats) at baseline (black) and dosed with 3 µM Terodiline (teal) are shown for a representative well.

Changes in rise time and triangulation were also readily quantified with LEAP. Astemizole (n = 6 wells per concentration) induced a dose-dependent increase in rise time (Fig. 6b) for Coyne hiPSC-derived cardiomyocytes, likely resulting from a relatively immature ion channel expression and the impact of hERG block on the resting membrane potential, as reported previously for hiPSC-derived cardiomyocytes^12,32,33^. The FP amplitude (AMP) was also computed from the same wells before and after dosing, with the percent change of AMP being sensitive to addition of Astemizole (Fig. 6c), such that FP AMP and LEAP rise time were each able to detect changes in cardiac depolarization. An example of reduced FP AMP is shown for a single electrode at baseline and 30 nM Astemizole (Fig. 6c, inset). However, at high doses, the FP AMP can become too small for detection, as indicated here by quiescent (Q) wells.

Terodiline caused a dose-dependent increase in triangulation for iCell CM^2^. Triangulation was assessed using the triangulation ratio^34^, such that a decrease in triangulation ratio indicates an increase in triangulation (1 = perfect “square”, 0.55 = perfect “triangle”). The triangulation ratio steadily decreased in a dose-dependent manner for Terodiline (Fig. 6e). Triangulation was particularly prominent at the highest dose (Fig. 6d), indicating arrhythmogenic risk, despite the absence of APD90 prolongation^34^. The change in repolarization was also assessed in the raw FP signals from the same wells, as illustrated by the example in Fig. 6f. An increase in triangulation was reflected by a broadened duration and reduced amplitude of the repolarization feature in the FP. The small FP repolarization feature in the presence of triangulation makes automated detection difficult, highlighting the utility of the LEAP signal for evaluating triangulation.

### LEAP captures important features of action potential morphology

**Figure 7:**
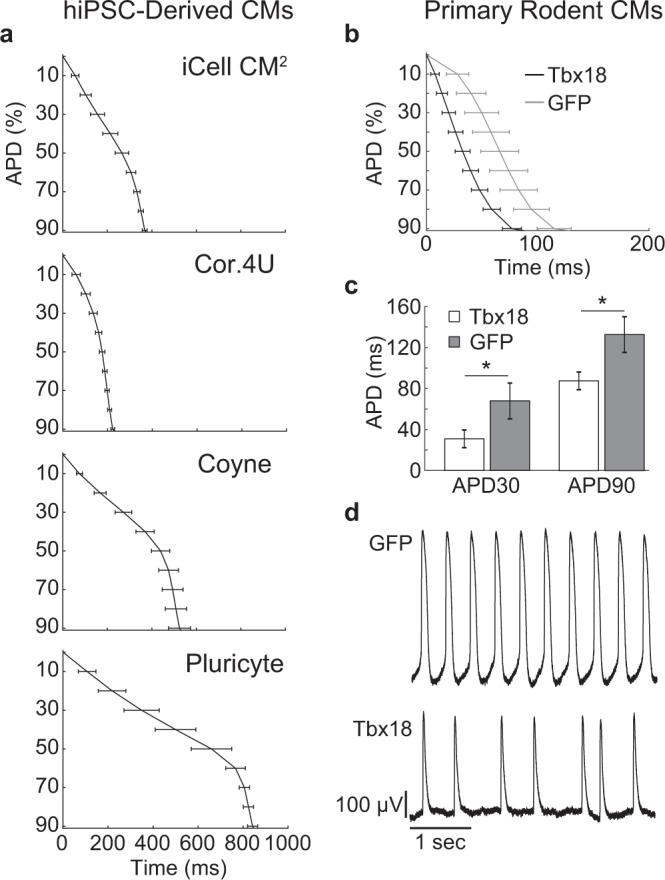
LEAP morphology from hiPSC-derived and primary rodent cardiomyocytes. (**a)** LEAP repolarization timing is shown for four sources of hiPSC-derived cardiomyocytes. Repolarization is represented as the mean ± standard deviation of APD across wells for APD0 to APD90 (iCell CM2 n = 20 wells, Cor.4U n = 17 wells, Coyne n = 24 wells, Pluricyte n = 5 wells). (**b)** LEAP repolarization timing is shown for GFP-transfected (n = 17) and Tbx18-transfected (n = 8) primary neonatal rat ventricular cardiomyocytes. (**c)** Tbx18 transfection altered AP morphology by decreasing APD30 and APD90 relative to GFP-transfection (Mann Whitney U-Test, APD30 p < 0.001, APD90 p < 0.001). (**d)** GFP-transfected cardiomyocytes exhibited more regular beating than Tbx18 (GFP BP CoV = 8.47 ± 8.50, Tbx18 BP CoV = 39.39 ± 29.23, p < 0.001).

In addition to enhancing the detection of compound effects, the large amplitude and high resolution of LEAP can be used to discern subtle differences in AP morphology across cell types and disease models. LEAP was successfully induced across four hiPSC-derived cardiomyocyte lines (Fig. 7a). Each panel of Fig. 7a shows the mean and standard deviation of APD from 10 to 90% across replicate wells for each cell type. The cell lines were characterized by different AP morphology, with Cor.4U having the shortest APD and Pluricyte the longest APD. Most cell lines exhibited lower interwell variability for APD during the late rapid repolarization phase, making APD90 a useful marker of repolarization timing. Coyne cardiomyocytes, however, were characterized by less variability during the plateau phase compared to repolarization.

**Figure 8:**
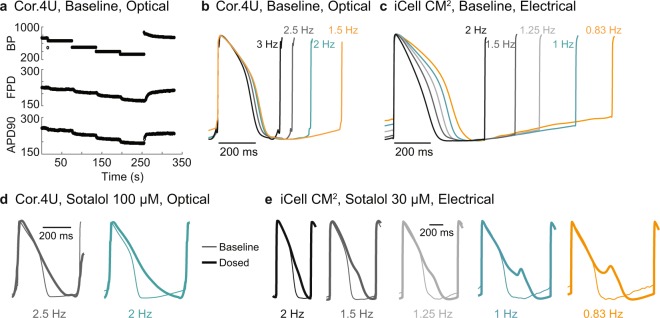
Optical and electrical pacing of LEAP signals can be used to control beat period and reveal rate-dependent effects. ChR2-transduced Cor.4U were paced using 5 ms blue light pulses at 1.5, 2, 2.5, and 3 Hz for 60 seconds each (**a**,**b**,**d**). Similarly, iCell CM^2^ cardiomyocytes were paced using a dedicated stimulation electrode (50 µA, 200 µs) at 0.83, 1, 1.25, 1.5, and 2 Hz (**c**,**e**). Both experiments were carried out on CytoView MEA 24-well plates. (**a)** BP, FPD, and APD90 are shown from one example well across the four rates. (**b)** Optically paced Cor.4U LEAP waveforms (averaged across 5 beats) from a representative well are shown at each rate. (**c)** Electrically paced iCell CM^2^ LEAP waveforms (averaged across 5 beats) from a representative well are shown at each rate. (**d)** When Cor.4U cardiomyocytes were dosed with 100 µM Sotalol, the AP was prolonged. Due to this prolongation, pacing at higher rates was not achieved. (**e)** Similarly, when iCell CM^2^ cardiomyocytes were dosed with 30 µM Sotalol, the AP was prolonged with greatest prolongation occurring at slower rates. EADs were present at slow rates, but not at faster rates.

LEAP was also induced on primary neonatal rodent ventricular myocytes (NRVM) and shown effective for distinguishing induced changes in AP morphology (Fig. 7b). Tbx18 is an embryonic transcription factor that has been shown to induce reprogramming of ventricular cardiomyocytes to specialized cardiac pacemaker cells^35–37^. As anticipated from a nodal-like AP, Tbx18-transduced NVRMs exhibited a shortened APD30 and APD90 compared to GFP controls (Fig. 8c, Mann Whitney U-Test, APD30 p < 0.001, APD90 p < 0.001). In addition, beat period coefficient of variation was significantly higher for Tbx18-transduced NVRMs compared to GFP-transduced (GFP BP CoV = 8.47 ± 8.50, Tbx18 BP CoV = 39.39 ± 29.23, p < 0.001). Notably, the NRVMs exhibited a more irregular spontaneous beat rate than commercial hiPSC-CMs (typically < 1%), as measured by beat period coefficient of variation^20^ (Fig. 7d). These differences may reflect the relative immaturity of the hiPSC-CM phenotype, as fetal cardiomyocytes exhibit an innate automaticity that is absent in adult cardiomyocytes^38,39^. The variable beat rate across cell types highlights the value of controlling beat rate for quantification of AP morphology across distinct cell populations.

Optogenetic and electrical stimulation were used to successfully modulate and control LEAP beat period across long periods of time, important for comparison across cell types and for allowing the detection of rate-dependent drug effects and cell behavior. To demonstrate optical pacing of LEAP signals, Cor.4U cardiomyocytes were transiently transfected with ChR2 via mRNA-based Xpress.4U LightPace Kit^40^. Cardiomyocytes were then paced with 5 ms pulses of blue light delivered at 1.5 Hz, 2 Hz, 2.5 Hz, and 3 Hz consecutively for 60 seconds each. Beat period adapted almost immediately, while FPD and APD90 slowly adjusted toward steady state over the course of 20–30 beats^20^. Figure 8a shows BP, FPD, and APD90 over the successive pacing rates for a representative well. Overlaid average LEAP beats from each of the pacing rates are presented in Fig. 8b.

Similarly, electrical pacing was performed in a separate experiment via a dedicated stimulation electrode included in the multiwell MEA plate. iCell CM^2^ cardiomyocytes were successfully paced at increasing pacing rates (0.83, 1, 1.25, 1.5, 2 Hz), with the average LEAP beats from each of the pacing rates presented in Fig. 8c. Note that slower rates were included as the spontaneous beat rate of the iCell CM^2^ was slower than that of the Cor.4U cardiomyocytes. Also, the AP morphology was qualitatively more sensitive to beat rate for the iCell CM^2^ than the Cor.4U cardiomyocytes, as highlighted by the difference in LEAP shape between 1.5 and 2 Hz for the two cell types.

Following baseline pacing, Sotalol (Cor.4U = 100 µM; iCell CM^2^ = 30 µM), a hERG blocker displaying reverse use-dependence, was added to the wells. LEAP induction was performed 20 minutes post-dose, and the pacing protocol was repeated at 30 minutes post-dose. As expected, Sotalol induced AP prolongation for both cell types (Fig. 8d,e) and pacing revealed that prolongation was greatest at slower beat periods. Indeed, EADs were observed at the slowest pacing rates for iCell CM^2^ cells, but increasing pacing rates eliminated the repolarization instabilities (Fig. 8e).

## Discussion

LEAP is the first high throughput, non-invasive, label-free method to capture AP morphology extracellularly from an intact cardiomyocyte syncytium. LEAP induction increases cell-electrode coupling, resulting in an AP morphology in the voltage signal recorded at the electrode from nearby cardiomyocytes for assay-relevant timescales.

LEAP induction differs from previous technologies for acquiring AP waveforms from microelectrode arrays, the majority of which utilized electroporation or 3D structures to augment the cell-electrode interface. Electroporation has been the method of choice^25,26,41^ for recent studies, with successful AP recordings acquired by opening holes in the cell membrane via high intensity electrical stimulation. However, the AP signals decay rapidly, requiring considerable effort in the analysis to compensate for changes in signal shape and amplitude^25^. In contrast, LEAP induction produced AP waveforms that were stable for 20 minutes, and often much longer. The continuing development of 3D microelectrodes has also demonstrated the ability to record stable action potential signals^23,29,41^, in some cases by encouraging cells to engulf the electrode. It remains to be seen if these processes can be adapted to higher throughput implementations.

A key advantage of LEAP for AP measurements is its compatibility with existing commercial solutions for medium-to-high throughput multiwell MEA platforms. As such, the LEAP assay benefits from the stable environmental controls of these platforms, the well-documented experimental protocols for FP recordings^4,17,18,20^, and the ability to directly link AP morphology to the existing literature of FP studies across a variety of applications^42–44^. The LEAP signal also allows the user to gather additional information from the same planar microelectrode arrays used in “gold standard” multiwell FP measures of cardiac electrophysiology. Currently, extracellular FPs are often used for high throughput assessment of cardiomyocyte electrophysiology and compound safety^4,16–18,20^. The large amplitude and high resolution of the LEAP signal enables automated detection of arrhythmic events, critical for medium-to-high throughput applications, that would typically require manual identification with FP assays. By measuring AP shape, LEAP also provides complementary metrics, such as APD, rise time, and triangulation, to those available with the FP (Fig. 6). As an example, FP AMP is extremely sensitive to changes in depolarization, but only provides information in a relative sense (e.g., change from baseline), whereas AP rise time provides a measure of depolarization rate in absolute units. Also, as seen in the FDA-initiated CiPA studies, the FP signal was often uninterpretable following strong hERG and sodium channel blockade^17,20^, but the effects on amplitude and repolarization are easily quantified via the LEAP signal. Similarly, while triangulation is qualitatively visible in the shape of the FP repolarization, it is difficult to quantify and can even obscure the repolarization feature. LEAP enables automation of these measurements for high throughput applications, moving towards a “plate-reader style” assay for cardiac risk, cell maturity, or disease assessments. Indeed, the majority of the experiments described in this manuscript were performed as endpoint assays, with a single LEAP measurement acquired, as compared to the traditional baseline vs. post-dose assays typical of FP studies.

Compound responses in hiPSC-derived cardiomyocytes can differ from those observed in human clinical trials due to differences in channel expression, maturity, and absence of other heart structures. For example, astemizole, a hERG blocker, induced a dose-dependent increase in rise time in this study, which is typically not observed in native adult cardiomyocytes. This difference likely reflects the immature ion channel expression. The hERG channel plays a more prominent role in the regulation of repolarization and diastolic potential in hiPSC-derived cardiomyocytes compared to primary adult cardiomyocytes, likely due to the relatively lower functional expression of Ik1^12,32^. As a result, blocking hERG leads to an overall depolarization of the membrane potential, thereby increasing the probability of sodium channel inactivation and reducing the rate of depolarization at the beginning of each action potential. Slowed rise time in response to other hERG blockers, such as E-4031 and Flecainide, has been reported in hiPSC-derived cardiomyocytes^12^.

Similarly, Verapamil, a calcium channel blocker, shortened APD as reported previously *in vitro*^16^, despite showing slight QTc prolongation in human clinical trials^45^. In the intact heart with spatially segregated sinoatrial and atrioventricular nodes, calcium channel blockade leads to delayed nodal depolarization. In contrast, in the mixed cardiomyocyte populations, such as hiPSC-derived cardiomyocyte monolayers, blocking calcium channels primarily impacts the plateau phase of the action potential, while sodium is the primary driver of depolarization and conduction^3,46^. Precisely because of these nuances with *in vitro* hiPSC-derived models, it is vital to carefully measure the spatiotemporal details of cell behavior, making LEAP a valuable tool for stem cell development and maturity assessments.

Automated patch clamp (APC) and various optical approaches have previously been reported for acquisition of AP signals at scale. APC devices have demonstrated the ability to measure ionic currents from expression systems^6^, with systematic comparisons to manual patch clamp recordings underway^47^. However, there are limited reports on the success of APC for recording directly from cardiomyocytes^7,8^, largely due to difficulties in achieving giga-ohm seal resistance which results in current rundown. Additionally, APC systems rely on cardiomyocyte suspension, thus removing the propagation of depolarization seen natively in cardiomyocyte networks and potentially leading to biased selection of cardiomyocytes for recording. By comparison, optical approaches are typically used to measure AP signals from intact cardiomyocyte monolayers^9,11,12,16^, either through introduction of voltage sensitive dyes^9^ or genetic expression of voltage sensitive proteins^10^. Unlike manual patch clamp, AP signals acquired optically are not in absolute voltage and instead must be normalized in amplitude, preventing the determination of resting membrane potential. LEAP signals are similarly normalized and compare favorably with previous reports for optical AP measurements^9^. However, unlike optical approaches, LEAP induction does not require the use of dyes or genetic modifications that may exert electrophysiological effects on the cells^13,14^ or distort the AP waveform^10^. Further, LEAP allows multiple simultaneous measurements in a well from the same syncytium, enabling evaluation of cell morphology heterogeneity within each syncytium^48^, as well as changes in conduction patterns or velocity.

The LEAP assay stands to enable and enhance numerous applications in stem cell development, disease modeling, drug discovery, and pharmacological safety. Despite rapid advances in stem cell technology, hiPSC-CM maturity remains an active area of investigation^49,50^ and will only improve the prospect of establishing patient-specific or disease-in-a-dish models of human biology. Importantly, careful characterization of features like rise time, prolongation, and triangulation are vital for characterizing the evolving boundaries on the ability of hiPSC-CMs to recapitulate mature human cardiac biology *in vitro*, with the methods described here providing a scalable approach. In addition, LEAP signals can be used to characterize cell behavior across disease models and to identify the electrophysiological consequences of genetic manipulations. As shown here, LEAP was able to distinguish both inherent and genetically-induced differences in AP shape across many cell types, including both hiPSC-derived and primary cardiomyocytes. Finally, there has been increased interest in 3D cardiomyocyte constructs for improving the maturity and accuracy of *in vitro* drug screening and disease modelling. LEAP may provide further insight into the influence of structural and environmental cues on action potential features, such as upstroke velocity and rise time^51^.

Overall, LEAP is a powerful tool for cardiac electrophysiology that can enable high throughput quantification of AP morphology to accelerate the development of stem cell models of healthy and diseased human biology, while improving the automation and accuracy of cell-based electrophysiology assays for drug discovery and safety testing. The additional and complementary information offered by LEAP stands to enhance and enable many new applications in cardiac biology research.

## Methods

### Cell culture

Four lines of human stem cell derived cardiomyocytes (Cellular Dynamics International [iCell CM^2^], Ncardia [Cor.4U and Pluricytes], and Coyne Scientific [Coyne]) were plated according to cell supplier recommendations. Commercial supplier specifications included surface coating procedures, cell density, media composition and volume per well, and time in culture required to mature cells. Coyne cardiomyocytes were differentiated from hiPSCs according to a well-established small molecule protocol involving the modulation of Wnt signaling pathways^52^. In all cases, fibronectin (50 µg/mL) was prepared in 1x sterile PBS and applied to the well using a droplet to ensure that the cells adhered over the microelectrode array. Additional protocol details for each commercial cell line are listed in Table 1. Protocols were designed to achieve an interconnected syncytium of cardiomyocytes, referred to elsewhere in the manuscript as simply cardiomyocytes. LEAP experiments were then performed approximately one to two weeks post plating.

**Table 1:** Cell plating protocol details.

Cell Type	Cell density per well	Fibronectin drop size	Incubation time	Days *in vitro* on MEA
iCell CM^2^	50,000	5 µL	1 hr	DIV 7
Cor.4U	25,000	8 µL	1 hr	DIV 7
Pluricytes	30,000	8 µL	3 hr	DIV 14
Coyne	75,000	8 µL	1 hr	DIV 7

This table provides the cell plating protocol details for all commercially-available hiPSC-CM lines.

In addition, neonatal rat ventricular myocytes (NRVMs) were used for a subset of studies according to methods described previously^36,53^. Experiments with rats were performed in compliance with the relevant laws and institutional guidelines and were approved by the IACUC of Emory University. Briefly, NRVMs were isolated from the lower third of the heart of 1–2 day old Sprague Dawley pups (Charles River Laboratories International, Inc) of both sexes, pooled across animals, and then plated at 210,000 cells per cm^2^ on CytoView MEA 24-well plates. One day post-isolation, NVRMs were transduced with either Ad-Tbx18-IRES-GFP or Ad-GFP. Tbx18 is an embryonic transcription factor that has been shown to induce reprogramming of ventricular cardiomyocytes to specialized cardiac pacemaker cells^35,36^. Experiments were performed 2–5 days post-transduction^36^.

### MEA Measurements

All cell types were seeded on multiwell MEA plates (Axion BioSystems, Inc.) and data were acquired using the Maestro Pro multiwell MEA platform (Axion BioSystems, Inc.). Data reported here were taken from a variety of MEA plate types, including Classic MEA 48- and 96-well plates and CytoView MEA 24- and 96-well plates, as noted in each legend. On the day of the experiment, the cell culture plate was moved directly from the incubator to the Maestro Pro for recordings. The device automatically adjusted and controlled the environment (37 °C and 5% CO_2_) to maintain temperature and media pH. Voltage data were sampled from 768 electrodes simultaneously at 12.5 kHz. The device acquired data with a bandwidth of 1 Hz to 2 kHz for FP only recordings, whereas LEAP recordings were acquired at 0.01 Hz to 2 kHz to faithfully measure the low frequency content of the AP waveforms.

LEAP induction was performed using the AxIS Navigator software (Axion BioSystems, Inc). LEAP induction operated independently on each electrode and involved delivery of an electrical stimulus to the selected planar microelectrodes on Axion MEA Plates (Axion BioSystems, Inc.) for 10 minutes, during which the MEA plate remained docked in the Maestro Pro. For all experiments in this study, the electrical stimulus was an alternating square wave (+/− 1 Volt, 100 nA, 8.33 kHz) delivered through the dedicated stimulator in the Maestro Pro for each selected electrode. Following the induction phase, cell coupling to the electrode was enhanced, such that AP signals were recorded as the voltage on the electrodes. The changes in coupling were also observed visually under bright field at 4x magnification (Supplemental Videos S1 and 2).

LEAP signals were quantitatively distinguished from FPs based on signal amplitude and variability. Because the LEAP signal deviates from zero for longer during each plateau phase, LEAP signals exhibit greater variability compared to field potentials. To be considered a LEAP signal, the signal must exhibit a standard deviation greater than 100 µV and an amplitude greater than 350 µV, although most LEAP signals are much larger (~ 1–10 mV). In addition, the largest and most robust LEAP signal in each well was selected for beat averaged overlays and analyses. Traces in the figures are unaveraged unless otherwise stated.

For comparison, electroporation was also used to induce AP signals on the microelectrodes using short, high intensity electrical stimuli via the Cardiac Pacing Block in AxIS Stimulation Studio (30 pulses at 1 Hz, 800 mV, 5 µA, and 4 ms duration) similar to previously published reports^25,26,54^.

### Experiment design

For all dosing experiments, the LEAP induction was started 20 minutes after administering the compounds to the plate, such that the LEAP measurements were acquired 30 minutes post-dose. When FP measurements were also acquired post-dose, the FP data was acquired from 15–20 minutes post-dose. In the majority of dosing experiments, the LEAP measurements were only made at 30 minutes post-dose, and the dosing conditions were compared to the vehicle control wells (see Figs 5 and 6). In Fig. 8, however, LEAP induction was performed on a subset of electrodes in each well in baseline and then in a separate subset of electrodes in each well after dosing. The LEAP signals from baseline and dosed conditions were overlaid in Fig. 8.

The compounds used in this study were Nifedipine (Sigma, cat. no. N7634), E-4031 (Cayman Chemical, cat. no. 15203), Verapamil (Sigma, cat. no. V4629), Astemizole (Sigma, cat. no. 1044301), Terodiline (Sigma, cat. no. T4577), and Sotalol (Sigma, cat. no. S0278).

### Data analysis

Three primary endpoints were derived from the cardiac FP in the baseline and post-dose conditions: (1) spike amplitude (AMP), (2) field potential duration (FPD), and (3) beat period (BP). To account for rate dependent effects, FPD was are also reported as beat rate-corrected (FPDc) using the Fridericia correction^55^. For these metrics, FP beats were automatically detected by AxIS Navigator and/or the CiPA Analysis Tool software (Axion BioSystems, Inc.), as described previously^20^. An example of the cardiac FP and the associated measurement definitions is shown in Fig. 1.

Analogous measures were derived from the cardiac LEAP signal: (1) rise time, (2) action potential duration (APD), and (3) beat period (BP). For these metrics, LEAP beats were automatically detected by AxIS Navigator and/or the CiPA Analysis Tool software (Axion BioSystems, Inc.). LEAP beats were then amplitude normalized from zero (trough) to one (peak), aligned at beat start defined as the max slope of the rising edge, and, in some cases, averaged across beats. Rise time is measured as the time between the take-off point to the peak of the LEAP (Fig. 1b, top inset). APD is measured from beat start to 30%, 50%, and 90% voltage repolarization (APD30, APD50, APD90, Fig. 1b, top), where full repolarization is defined from peak to trough. BP is defined as the time between two consecutive beat starts.

In addition, key determinants of arrhythmogenic risk and AP morphology were also quantified from the cardiac LEAP signal. Triangulation, which is a measure of repolarization rate, was quantified by the triangulation ratio, defined here as the ratio of APD50 to APD90^34^. A decrease in the triangulation ratio indicates an increase in triangulation of the repolarization phase, leading to increased risk of arrhythmic events^56,57^. Similarly, arrhythmic events, such as early after depolarizations (EADs), can be reliably detected in an automated high-throughput fashion (see Fig. 4d). LEAP EADs were defined as peaks between depolarization and repolarization with an amplitude of at least 20 µV and greater than 2.5% of the LEAP amplitude^3^.

Statistical analyses were performed in Matlab 2018a (The Mathworks, Inc.) or RStudio 3.3.1 (The R Foundation for Statistical Computing). For comparison of group means, the Shapiro Wilk test was applied to test the normality of the data distribution and the F-Test was applied to test for equal variances. If either assumption was violated, the appropriate non-parametric tests were used to compare means. Linear correlations were considered significant if the slope of the best fit line was significantly different from zero. All statistics were considered significant at p < 0.05, and p-values < 0.001 were reported to that precision.

### Pacing

For optical pacing, Cor.4U cardiomycytes plated on a CytoView MEA 24-well plate were transiently transfected on DIV5 with ChR2 via mRNA-based Xpress.4U LightPace Kit from Ncardia^40^. At DIV6, cells were paced using 5 ms pulses of blue light (470 nm) from a multiwell light delivery device (Lumos, Axion BioSystems, Inc.). For electrical pacing, iCell CM2 cardiomycytes plated on a CytoView MEA 24-well plate were electrically paced via the dedicated stimulation electrode in each well and the Cardiac Pacing Block with default settings (800 mV, 5 µA, 200 µs) in AxIS Navigator.

## Supplementary information

Supplemental Video 1

Supplemental Video 2

## Data Availability

The datasets generated during and/or analyzed during the current study are available from the corresponding author on reasonable request.
